# Ablation of Paroxysmal Atrial Fibrillation: between Present and Future

**DOI:** 10.31083/j.rcm2504140

**Published:** 2024-04-08

**Authors:** Antonio Gianluca Robles, Zefferino Palamà, Antonio Scarà, Alessio Borrelli, Domenico Gianfrancesco, Francesco Bartolomucci, Martina Nesti, Elena Cavarretta, Gabriele De Masi De Luca, Silvio Romano, Luigi Sciarra

**Affiliations:** ^1^Department of Life, Health and Environmental Sciences, University of L'Aquila, 67100 L'Aquila, Italy; ^2^Cardiology Department, Ospedale “L. Bonomo”, 76123 Andria, Italy; ^3^Electrophysiology Unit, Casa di Cura “Villa Verde”, 74121 Taranto, Italy; ^4^GVM Care and Research, Ospedale San Carlo di Nancy, 00165 Rome, Italy; ^5^Cardiology Unit, CNR Fondazione Toscana “Gabriele Monasterio”, 56124 Pisa, Italy,; ^6^Department of Medical-Surgical Sciences and Biotechnologies, Sapienza University of Rome, 04100 Latina, Italy; ^7^Cardiovascular Department, Mediterranea Cardiocentro, 80122 Naples, Italy; ^8^Department of Cardiology, Ospedale Panico, 73039 Tricase, Italy

**Keywords:** paroxysmal atrial fibrillation, catheter ablation, rhythm control

## Abstract

Pulmonary vein isolation (PVI) is the established cornerstone for atrial fibrillation (AF) ablation, indeed current guidelines recognize PVI as the gold standard for first-time AF ablation, regardless of if it is paroxysmal or persistent. Since 1998 when Haïssaguerre pioneered AF ablation demonstrating a burden reduction after segmental pulmonary vein (PV) ablation, our approach to PVI was superior in terms of methodology and technology. This review aims to describe how paroxysmal atrial fibrillation ablation has evolved over the last twenty years. We will focus on available techniques, a mechanistic understanding of paroxysmal AF genesis and the possibility of a tailored approach for the treatment of AF, before concluding with a future perspective.

## 1. Introduction

Atrial fibrillation (AF) is classified as a sustained arrhythmia, which has the highest prevalence in the adult population with more than 6% in those over 65 years old having the condition. It is expected to grow in future given the increasing population longevity and expansion of opportunistic and systematic AF screening [1]. AF is associated with an increased risk of stroke, heart failure and mortality and for these reasons it must be intercepted and treated following the “ABC” scheme suggested by the latest ESC guidelines [1]. Apart from prognosis improvement, AF treatment also aims to lead to better symptom control and catheter ablation is currently a well-established weapon for symptomatic, drug-refractory AF, to a different extent depending on its type [1]. Current guidelines differentiate AF as paroxysmal, persistent (short- and long-standing) and permanent, based on temporal arrhythmic behaviour criteria, regardless of its mechanism [1]. For sure this differentiation impacts treatment strategies, indeed permanent AF has no space for ablative treatment unless the patient is a candidate for ablate and pace [1]. Conversely, the best impact of ablation is on paroxysmal forms. In 1999 Haïssaguerre was the pioneer of AF ablation demonstrating how segmental ablation at the pulmonary vein (PV) ostia reduced AF burden in the follow-up [2]. At the basis of this approach was the discovery of the key role of the PV’s muscular sleeves’ firing activity in triggering AF. Over the next two decades, PV isolation became the gold standard of AF ablative treatment and we saw a vivid and rapid improvement in techniques and technologies aiming to a durable and, at the same time, safer and faster PV lesions. Moreover, during this period we testified also a growing enthusiasm toward AF ablation supported by trials (CABANA and its substudies, and EAST-AFNET4) demonstrating that rhythm control—especially when achieved by ablation—improves outcomes over the only rate control and thus refusing the previous dogma according to which there were no differences in outcomes between rate and rhythm control (AFFIRM) [3, 4, 5, 6, 7, 8, 9]. Clinical evidence is also going towards the demonstration that the best rhythm strategy control is ablative and not pharmacological, above all if performed as soon as possible, in an early stage to avoid the onset of an irreversible and self-feeding atrial cardiomyopathy. This concept led to another dogma that should be discussed: “AF begets AF” [10]. Not all the paroxysmal AF (PAF) forms evolve through more persistent ones, and this depends on the complex interplay of genetics, risk factors, underlying cardiomyopathy, comorbidity and mechanism of AF induction: patients with only PAF—the so-called “Lone AF”—rarely go toward the development of persistent AF (PeAF) [11]. The former is typically seen in young patients, without any disease and in whom triggers—sometimes represented by synchronized supraventricular tachycardias (SVTs)—are the only determinants [12, 13].

## 2. A Mechanistic Approach to Paroxysmal AF Ablation

**Fig. 1.**
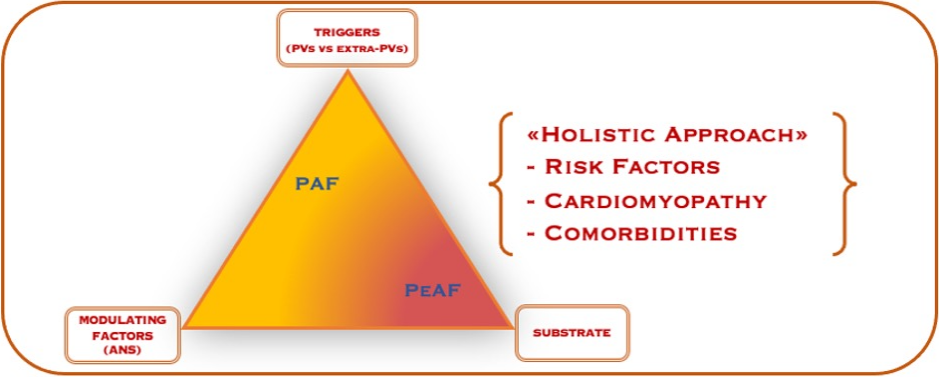
**Paroxysmal AF pathophysiology**. The Coumel’s triangle shows the interplay of triggers, modulating factors and substrate in the genesis and perpetuation of atrial fibrillation whose treatment should consider a “holistic approach” aiming to correct risk factors, cardiomyopathy and comorbidities. PVs, pulmonary veins; PAF, paroxysmal atrial fibrillation; PeAF, persistent atrial fibrillation; ANS, autonomic nervous system.

AF represents the paradigm of Coumel’s triangle, a shared theory since 1960 that explains the genesis of cardiac arrhythmias through the variable relationship between triggers, arrhythmogenic substrate and modulating factors [14]. In particular, triggers play a key role in PAF, but, as we will see later, also modulating factors like autonomic tone impact its initiation and perpetuation [12] (Fig. 1).

The best recognized AF trigger is represented by PV firing activity. In his milieu paper considering 45 patients suffering from PAF, Haïssaguerre demonstrated: (1) the highest prevalence (95%) of ectopic beats originating from PVs and, (2) no AF recurrences in 62% of the sample after ablation targeted to ectopic sources in PVs. This opened the era of pulmonary vein isolation (PVI) which started from a segmental PV’s ablation aiming at the abolition of near-field muscular sleeves’ electrograms recorded by a circular catheter (LASSO) based on a fluoroscopic approach [2]. Later, AF ablation moved toward a more extensive ablation aiming to complete electrical isolation of all PVs, which was earlier more ostial but later it became a wider antral encircling [15]. The latter prevents PV’s stenosis and subsequent development of pulmonary hypertension and at the same time guarantees PVs’ electrical isolation and a localized substrate and autonomic modification, resulting in lower arrhythmic recurrences [15]. Indeed, antral regions often host complex fragmented atrial electrogram (CFAE) – a source of micro-reentry especially in PeAF – and parasympathetic ganglia and nerve fibers [15].

Even if PVs are the more prevalent trigger for PAF, we could recognize other extra-PVI sources. These may be ectopic atrial beats arising from superior vena cava, crista terminalis, fossa ovalis, coronary sinus or vein of Marshall and determining focal or reentry atrial tachycardias triggering AF [16, 17]. Even macro-reentry tachycardias like atrial flutter (typical or atypical), atrio-ventricular node reentry tachycardia and atrio-ventricular reentrant tachycardia may trigger AF [18, 19]. There is consistent data in literature supporting a “*tailored approach*” targeting only extra-PVI triggers for AF ablation, especially in younger patients, with a long history of palpitations and without structural heart disease nor cardiovascular risk factors [12, 13, 19]. This approach, limited to a well-selected patients’ category, is demonstrated to be effective because it eliminates the AF-triggering arrhythmia with a simpler and faster procedure: the best example is a slow pathway ablation in a patient with atrioventricular nodal reentrant tachycardia (AVNRT) degenerating in AF. A *tailor-based* method may seem time-consuming because of the need of a detailed electrophysiological (EP) study, but we have to bear in mind that a time invested for an EP study may be useful to avoid an unnecessary longer PVI procedure [13, 19]. Clinical history and analysis of stored traces from external recording systems or implantable devices add precious clues for the better clinical depiction of AF episodes [12].

Modulating factors like autonomic tone impact AF genesis [20]. Indeed, it alters the electrical properties of atrial myocytes and this translates into complex and not always predictable effects on triggers and substrates, thus affecting not only the temporal behavior of AF (paroxysmal vs persistent) but also the arrhythmic burden and heart rate, therefore impacting on patient quality of life and heart failure risk [20, 21]. Given this deep influence of the autonomic nerve system on AF, a working group suggested a new and revised pathogenetic hypothesis called “autonomic Coumel’s triangle” instead of just the Coumel’s triangle concept [21].

Based on this knowledge, there is a growing interest and scientific evidence about the treatment of vagal-mediated AF with just cardioneuroablation instead of PVI [22, 23, 24, 25]. Conversely, there are patients suffering from episodes of PAF clearly mediated by adrenergic over-stimulation like physical or emotional stress involved in competition [14, 26, 27]. This data justifies the results of the study by Capucci *et al*. [28] according to which the best pharmacological therapy for rhythm control is the combination of flecainide and metoprolol.

## 3. Energy Sources for AF Ablation

As already stated in the text, PVI is the cornerstone of ablation for patients with symptomatic, drug-refractory AF, regardless of its type [1]. This is well established by current guidelines and it can be indifferently performed utilizing point-by-point radiofrequency (RF) or single-shot devices [1, 29]. The latter have been designed to fit PVs and, so, they are primarily born to be employed in first-time AF ablation procedures with the main aim of reducing the duration of the procedure as much as possible by sewing it back to a purely anatomical ablation. Available sources may be summarized as follows: RF, cryoenergy, laser, ultrasound and pulsed-field ablation (PFA) [30].

Going back to the history of AF ablation, the first seminal experience was the work of Haïssaguerre—previously discussed—consisting of a fluoroscopy-guided segmental RF ablation of PVs’ ostia [2]. Later, with the advent of 3D-mapping systems, Pappone *et al*. [31, 32] were the first to perform PVI with the aid of RF catheters provided with magnetic sensors in order to achieve a complete ostial encircling without the use of fluoroscopy.

RF still today remains the more employed energy source for AF ablation procedures [30]. It is the most studied and updated technology over the years. Indeed, it passed from non-irrigated catheters used in temperature-controlled mode to irrigated catheters used in power-controlled mode (20–40 Watts) in order to reduce thrombo-embolic complications [33]. Power-controlled catheters improved with contact-force sensors which allowed to define parameters like Ablation Index (AI, Biosense Webster) and Lesion Size Index (LSI, Abbott) as surrogate of lesion quality and, at the same time, safety [34, 35, 36, 37, 38, 39]. Attempts to increase contact force, and at the same time safety, also were made by the introduction of remote catheter navigation. There were different experiences with variable results using both robotic and magnetic navigation [40, 41]. The use of 3D-mapping systems enabled with auto-tagging algorithms led us to the current workflow for PVI: the CLOSE protocol [42, 43, 44, 45].

The different companies updated their catheters to improve safety and efficacy by the possibility to deliver higher power and ensure better tip-tissue stability, not only by contact-force sensors but also by new tip features (e.g., TactiFlex, Abbott) [46, 47]. In this context, irrigated catheters were equipped with tip thermocouples in order to get tissue/tip temperature feedback and thus achieve a real “temperature-controlled” RF delivery (Qdot Micro, Biosense Webster, and DiamondTemp, Medtronic). Finally, the point-by-point RF delivery evolved increasingly moving towards greater speeding up of the procedure: protocols based on the delivery of high-power/short-duration and very high-power/very short-duration are included in this context [48, 49, 50, 51].

Taken together, point-by-point RF ablation evolved in a safe, fast (more or less 1 one-hour duration procedure is achievable) and effective ablative strategy with up to 98% first-pass isolation and 90% durability at 1 year [30]. To date, RF linear ablation has added values over the next discussed technologies since in particular, it ensures to approach and perform substrate modification in PeAF and complete fluoro-less AF ablation [52, 53].

The discussion about RF ends with the citation of the available single-shot tools which after a first unsatisfactory experience with circular catheters (PVAC®, Medtronic; and nMARQ™, Biosense Webster) are now generating new enthusiasm with balloon catheters (Toray-Satake balloon, Toray Industries; Heliostar, Biosense Webster; and Luminize, Boston Scientific) [54, 55, 56, 57, 58].

Among the single-shot tools available for sure the most important is represented by cryoballons, in use since 2003 [59]. It now has a consolidated reputation and studies that support its non-inferiority to RF, so much so that since 2016, the guidelines indicate it as the first choice in AF ablation, as an alternative to RF, based on preference and capabilities of the operator [29, 60, 61]. Compared with RF, cryoenergy seems to be less burdened by pericardial complications, it has no incidence of atrio-esophageal fistula but it has a greater incidence of phrenic nerve palsy, generally transient [30]. Currently, there are two available cryoballon systems on the market: Arctic Front Advance Pro, which is the 4th generation and newest Medtronic Cryoballon available in two different diameters (23 mm and 28 mm); and the more recent POLARx FIT, which is the second generation Boston Scientific Cryoballon and it has an adjustable diameter between 28 to 31 mm and an increased deflection angle of the sheath (155° against 135° of the Arctic Front ballon) [30, 62]. The list of cryoenergy tools available ends with the ultra-low temperature cryoablation (ULTC, Adagio Medical) linear catheter whose shape can be modified by preformed stylets, and which may produce deeper lesions but, at the same time, carries a greater risk of collateral damage [63].

Doubtless, the current main topic of AF ablation is PFA since its clinical introduction in 2018 [64]. It is a nonthermal energy that produces irreversible tissue damage by electroporation following the application of short-duration high voltage electrical fields [65]. To date, there are different companies carrying on clinical experimentation on their catheters with different footprints (linear vs oneshot tools) and, so, different PFA delivery modalities [30, 66]. PFA should spare collateral structures thus avoiding esophageal, phrenic and pericardial damages – as preclinically tested on non-human models – but coronary artery spasm has been reported with one available tool, and, recently, rare cases of acute kidney injury secondary to haemolysis after a large amount of erogation has been reported in one trial [67, 68, 69, 70, 71, 72, 73, 74, 75]. Given its myocardial selectivity, it is currently not suitable for cardioneuroablation [76]. Even if head-to-head large prospective, randomized, double-blind trials comparing PFA to other thermal sources for AF ablation do not currently exist, three multicenter studies (PULSED AF Pivotal trial; EU-PORIA; and ADVENT) have overall shown a good safety profile of PFA, excellent acute success with shorter duration procedure times and good long-term efficacy which is consistent with other established thermal energies [66, 77, 78, 79, 80].

Table 1 summarizes the main features of the three mainly employed energy sources: RF, cryoenergy and PFA.

**Table 1.** **Comparison of main energy sources technology features**.

	RF	CRYO	PFA
Available catheters footprint	- Linear (point-by-point)	Ballon	Different footprints
	- Ballon		
First pass PVI (%)*	98%	>98%	∼100%
Durability at 1 year (%)*	70–90%	60–73%	70–90%
Pericardial injury	+	Rare	NO
Aesophageal fistula	+	NO	NO
Phrenic nerve palsy	rare	+, usually transient	NO
Coronary artery injury	possible	NO	+, spasm only described with pentaspline
Hemolisys	NO	NO	possible
3D mapping system integration	YES	NO	ongoing
Zero-fluro	YES	NO	NA
Ablation beyond PVs	YES	NO	possible

*These data refer only to paroxysmal atrial fibrillation ablation. NA, not applicable; RF, radiofrequency; CRYO, cryoenergy; PFA, pulsed-field ablation; PVI, pulmonary veins isolation; PVs, pulmonary veins.

At the end of this chapter, endoscopic laser balloons and high-intensity focused ultrasound (HIFU) also deserve a brief mention. The former represents a promising single-shoot tool that showed similar freedom from AF and good long-term results in a head-to-head comparison with RF. Conversely, HIFU carries several limitations including difficult-to-achieve PVI and the significant burden of periprocedural complications [81, 82, 83, 84].

Table 2 (Ref. [60, 61, 77, 81]) provides a synopsis of the available trials comparing head-to-head the different energy sources.

**Table 2.** **Principal trials comparing head-to-head the different energy sources available for AF ablation**.

Trial name	Trial type	Energy compared	N. patients	Results
Kuck *et al*. [60] (FIRE AND ICE)	Multicenter	Cryoballon vs RF (power control)	762	Efficacy: CBA non inferior to RF.
	Randomized		(378:384)	Safety: no difference.
	(non-inferiority design)		PAF only	
Andrade *et al*. [61] (CIRCA DOSE)	Multicenter	4-min or 2-min Cryoballon vs RF (contact force)	346	No difference in 1 year efficacy (time to first recurrence and burden reduction assessed by ILR). Less fluoroscopy time for RF.
	Randomized		(115:116:115)	
			PAF only	
Reddy *et al*. [77] (ADVENT)	Multicenter	PFA vs thermal ablation (RF or CBA)	607	PFA non inferior to thermal ablation in regard of a composite endpoint of efficacy and device- and procedure-related seriuos complications.
	Randomized		(305:302)	
	(non-inferiority design)		PAF only	
Schiavone *et al*. [81]	Prospective two-arm nonrandomized propensity-matched observational	Laser ballon vs CBA	110	No difference in arrhythmia autcomes assessed by ILR.
			(55:55)	
			PAF 57.3%	No difference in procedure or fluoroscopy time.

RF, radiofrequency; CBA, cryoballon; PAF, paroxysmal atrial fibrillation; ILR, implantable loop recorder; AF, atrial fibrillation.

## 4. Future Perspectives and Conclusions

**Fig. 2.**
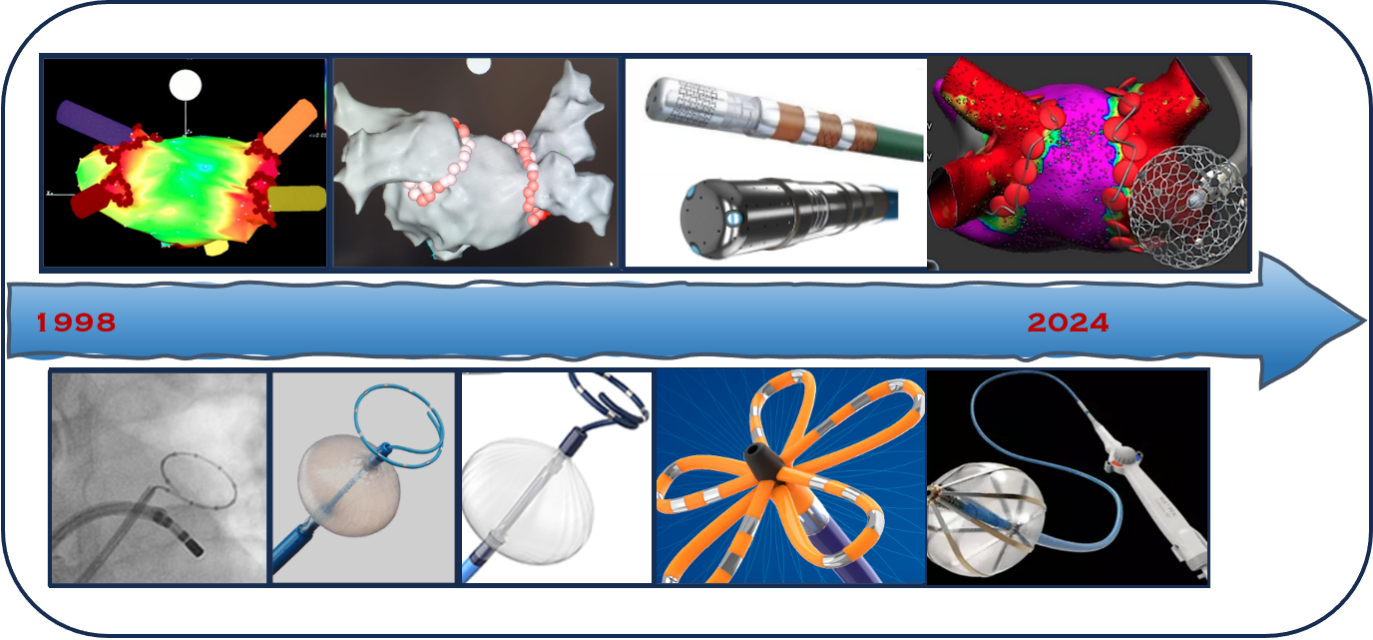
**Progress in paroxysmal AF treatment**. Starting from 1998, the timeline shows a schematic overview of strategies and technologies developed in the past 2 decades and ongoing for AF ablation. AF, atrial fibrillation.

This overview testifies how AF ablation is a vibrant research field. Specifically speaking about PAF, knowledge improvement is nowadays more directed toward new ablation technologies than a better understanding of AF initiation (Fig. 2). To this extent, we can defiantly say that PAF ablation is at a steady state and the future has already arrived. Indeed, an improvement in mapping strategies (high-density mapping with new multipolar catheters and integration with data acquired from sophisticated 3D-mapping systems tools and/or from cardiac computed tomography (CT) o cardiac magnetic resonance (CMR) imaging) are matter of development for PeAF ablation treatment, without a significative impact on PAF [12, 30]. For the latter, we all know the key role of PVs that are the target for the ablation whatever the energy is used. In this context, companies are improving catheter performance to achieve PVI as fast as possible but at the same time in a more safe and durable way. In this scenario, PFA is currently the leading tool, and we expect the most from it in the future. We expect a better knowledge of PFA and its integration in 3D-mapping systems (e.g., FARAPULSE™+RHYTHMIA HDx, Boston Scientific; VLCC/PFA generator+CARTO 3, Biosense Webster; and VOLT™, Abbott) in order to reduce or remove fluoroscopy and at the same time deliver precise point-by-point energy by tagging lesions, looking at gaps, and also the possibility to switch between PFA and RF (e.g., AFFERA™ Medtronic) [85, 86]. To this extent, intracardiac echography (ICE) may allow us to reduce or even abolish the use of fluoroscopy for PVI via PFA, as recently reported in a small case series [87]. On the other hand, ICE already allows complete zero-fluoro AF ablation via point-by-point RF [53, 88].

Notably, we must not forget that not all PAFs are PVs driven: clinical history and arrhythmic episode recordings (when available) may suggest extra-PV triggers or a vagal-mediated AF deserving a tailored ablative approach, beyond PVI, in a selected patient category [12]. This last point is not trivial. Maybe a better knowledge of the pathogenesis of PAF and an improvement in the available technologies—capable of guaranteeing at the same time short procedural times, effectiveness and safety, both for the patient and for the EP lab staff—will lead to AF ablation being first line treatment in future. In fact, it seems that the guidelines are moving in this direction, so much so that the current European and American guidelines have upgraded the class of recommendation for AF ablation, supported by trials according to which rhythm control through ablation—especially if early—rather than with antiarrhythmic drugs, improves outcomes [1, 89].

Finally, even if paroxysmal, AF should be understood as a chronic condition whose treatment is not limited to ablation alone, but possible risk factors, comorbidities and underlying cardiomyopathies must be treated according to the so-called holistic approach [1].
